# SANE (eaSy gAit aNalysis systEm): real-time dual-depth, marker-less system characterisation and robustness of gait parameters

**DOI:** 10.3389/frobt.2026.1691113

**Published:** 2026-05-15

**Authors:** Daniele Cafolla, Betsy D. M. Chaparro-Rico

**Affiliations:** Faculty of Science and Engineering, Intelligent Robotics Group, Department of Computer Science, Swansea University, Swansea, United Kingdom

**Keywords:** biomechanics, dual-depth, gait analysis, marker-less systems, movement analysis

## Abstract

Gait analysis is essential in clinical evaluation, biomechanics, and rehabilitation, yet conventional approaches often rely on complex marker-based systems that limit accessibility and real-time feedback. The SANE (eaSy gAit aNalysis systEm) platform was launched in 2020 and has previously been evaluated in multi-subject laboratory and clinical studies, where it demonstrated agreement with gold-standard marker-based systems for core spatiotemporal parameters and achieved high test–retest, inter-rater, and intra-rater reliability. Building on this validated foundation, the present work provides a system characterisation and robustness analysis of the latest dual-depth, real-time SANE implementation. The updated SANE system enables real-time extraction and visualisation of an expanded set of spatiotemporal and angular gait parameters—including gait speed, step and stride length, cadence, step and stride time, step width, foot angles, double support, and gait phase durations—using two depth cameras and AI-based pose estimation. A total of 80 walking trials performed by a healthy adult (39 years, 1.74 m, 73.0 kg) were acquired across four sessions over 1 week, with complete system shutdown and restart between sessions to rigorously challenge operational stability. Session-level means, within-session standard deviations, and Relative Error Measurement (REM%) between sessions were used to quantify robustness and session-to-session variability. Across all four sessions, gait outputs remained within published normative ranges for healthy adults. REM% values for primary spatiotemporal parameters were consistently below or around 5%, and standard deviations were low and comparable to values reported for marker-based systems, indicating stable, repeatable measurements over repeated restarts and days. Real-time computation and visualisation at frame rates up to approximately 80 frames per second further distinguish this system from traditional post hoc workflows. These findings characterise the operational robustness and real-time capabilities of the dual-depth SANE system in a controlled, single-participant setting and support its use as a practical, marker-less gait assessment tool in clinical and research environments, while motivating future studies on larger and pathological cohorts.

## 1 Introduction

The human gait cycle, characterized by a series of coordinated movements and phases, can be influenced by numerous factors, including neurological, orthopedic, and pathological conditions. Gait analysis is a critical area of research, particularly for understanding human locomotion and diagnosing various health conditions. Traditionally, gait analysis has relied on motion capture technologies, including systems that utilize physical markers. However, these systems can be expensive and cumbersome, restricting their accessibility and practicality in both clinical and everyday environments.

Clinical gait analysis often involves sophisticated equipment that can be expensive, requiring insurance or access to specialized facilities. While systems such as optoelectronic and 3D motion capture setups offer detailed biomechanical data, they have limitations regarding mobility and setup readiness (Cimolin, et al., 2022; Healy et al., 2018).

Recent advancements aim to address these limitations through the development of marker-less gait analysis systems to improve diagnostic capabilities and efficiency. For instance, inertial measurement units (IMUs) and computer vision techniques promise for real-time analysis while being more cost-effective and non-invasive compared to traditional systems (Ekanayake et al., 2015; Yeo and Park, 2020; Albuquerque et al., 2021). Systems employing IMUs can extract spatiotemporal metrics with significant accuracy, facilitating real-time monitoring during rehabilitation. Moreover, marker-less systems can capture gait dynamics without the need for bulky physical markers, making them more user-friendly for the general population.

In addition, other technologies using marker-less systems have emerged as viable alternatives, utilizing depth cameras and AI algorithms to derive gait metrics similarly to marker-based methods, yet with reduced complexity and cost. Research indicates differences in factors affecting traditional and AI-driven methods, including speed, cost, and setup burden (Chen et al., 2011; Hori et al., 2020). Traditional systems may necessitate significant preparatory time and alignment adjustments, while these automated systems can function seamlessly in more dynamic environments.

Key innovations in automated gait analysis include camera depth technology, which enhances spatial awareness and precision in capturing movement parameters. This technology offers depth perception and accuracy in determining gait kinetics, which may enable assessments of gait abnormalities over time. Recent studies demonstrate a strong correlation between AI-enhanced methodologies and the validity of measuring gait dynamics when compared to traditional methods in clinical settings (Imoto, Hirano et al., 2022). Furthermore, AI algorithms in analysing gait data have shown promise in reducing human error and facilitating rapid diagnostics across diverse populations, including patients recovering from strokes and those with Parkinson’s disease (Nakashima et al., 2025; Davey et al., 2024).

One challenge with current gait analysis methodologies is the inability of many systems to provide real-time processing without sacrificing accuracy. Traditional methods often rely on *post hoc* analysis, delaying necessary corrective interventions for patient improvement. However, new systems are beginning to offer solutions by streamlining data acquisition and analysis, enabling clinicians to evaluate and respond to patient needs more effectively and timely (Hulleck et al., 2022). These developments reflect a significant shift in the paradigm of gait analysis, moving from reactive assessments to proactive preventive care through enhanced accessibility to gait monitoring technologies.

Additionally, there is growing recognition that low-cost systems must maintain analytical accuracy comparable to high-end traditional models. Recent efforts have focused on validating the performance metrics of affordable systems against established benchmarks, such as those provided by optoelectronic laboratories, which helps in getting confidence in the adoption of accessible technology in outpatient and home care settings (Adhaye et al., 2023; Nong, et al., 2021).

The integration of technologies in gait analysis has led to the exploration of novel methodologies aimed at enhancing the reliability and validity of assessments. The expanding capabilities of automated gait analysis systems, particularly those employing AI and marker-less techniques, present exciting possibilities for clinical applications. However, to ensure that such emerging technologies match the accuracy and reliability standards of traditional marker-based systems, comparative validation studies are crucial.

The use of marker-based systems often deemed the “gold standard” in gait analysis, serves as a benchmark against which novel systems can be validated. Marker-based analysis provides high levels of detail in capturing spatiotemporal parameters and kinematics, making it essential for clinical assessments of gait disorders and pathologies. Nevertheless, the challenges associated with marker placement, setup time, and patient comfort have bolstered the need for more pragmatic solutions that retain diagnostic efficacy while alleviating the constraints of traditional methods (Chaparro-Rico and Cafolla, 2020; Sipari et al., 2022).

Chaparro-Rico and Cafolla’s study (2020) evaluates the reliability metrics of the SANE (eaSy gAit aNalysis system) across test-retest, inter-rater, and intra-rater tests; an approach that underscores the importance of establishing the robustness of new systems against traditional benchmarks.

Moreover, Sipari et al. (2022) presented an analytical perspective on the comparative performance of AI-assisted methodologies against traditional gait metrics through the lens of an improved SANE system with embedded AI. Their findings suggest that SANE’s spatiotemporal parameters exhibit a strong correlation with those obtained through conventional motion capture systems, supporting its applicability in clinical settings. By contrasting the gait parameters derived from different motion tracking technologies, they demonstrate the potential for AI-driven systems to achieve results comparable to gold standard methods.

Additionally, the importance of validation in real-time gait analysis cannot be overstated. Traditional systems require meticulous setup and can introduce variability based on the human operator’s expertise and the circumstances of data collection. As gait assessment technologies evolve toward rapid processing and improved user experience, validation against established techniques remains essential. Automated systems must not only analyse gait in real-time but also demonstrate their accuracy and reliability by offering results analogous to those gathered from traditional systems. This validation process involves comprehensive studies focusing on parameters such as step length, cadence, gait speed, and variability, which are critical for diagnosing various gait pathologies.

Furthermore, the challenges associated with reliance on traditional methods, especially in fast-paced clinical environments, make the validation of automated systems even more pertinent. By employing rigorous testing conditions and comparative analyses, emerging technologies can substantiate their claims of efficacy and reliability, ensuring a smoother transition from diagnostics to meaningful interventions.

The SANE (eaSy gAit aNalysis systEm), as presented in this paper, aim to bridge these gaps by introducing real-time analysis capabilities while expanding the range of gait parameters measured compared to prior versions introduced in earlier works (Chaparro-Rico and Cafolla, 2020; Sipari et al., 2022). By building on an already validated framework, SANE incorporates these new parameters and real-time analysis features, significantly enhancing its diagnostic accuracy and rehabilitative potential. Rigorous experimental campaigns have been conducted, and the results are presented and discussed, demonstrating the system’s improvements and potential. This positions the current work as a system-level characterisation and robustness study rather than a new multi-participant clinical validation.

While Cimolin, Healy, Ekanayake, Yeo, Albuquerque, Chen, Hori, Imoto, Nakashima, Davey, and others have reported marker-less or AI-based real-time gait analysis solutions, SANE uniquely integrates dual-depth imaging for enhanced spatial accuracy, automated real-time extraction of an expanded parameter set, and rigorous, session-to-session reliability analyses validated both in laboratory and clinical practice, as previously established by Sipari et al. (2022).

The system at the core of this study, SANE (eaSy gAit aNalysis systEm), was originally developed to provide an automated, marker-less alternative to the labor-intensive and time-consuming workflow of gold standard marker-based gait analysis systems. SANE uses dual-depth cameras and AI-driven real-time processing to extract a comprehensive set of gait parameters (Chaparro-Rico and Cafolla, 2020; Sipari et al., 2022). In our previous publications, SANE was validated in both laboratory and clinical settings, demonstrating robust agreement with marker-based systems for spatiotemporal measurements and high test-retest and inter/intra-rater reliability. The present paper builds on this validated foundation, advancing SANE through additional technical improvements, a broader set of real-time parameters, and strict robustness testing under realistic clinical and laboratory conditions. Dual-depth is preferable in this study because it directly reduces occlusions and stabilizes 3D joint estimates in ways a single depth camera or IMUs alone cannot. With two depth cameras calibrated into a common 3D frame and fused per frame, SANE can still track feet and ankles when one view is partially blocked, avoiding the landmark dropouts that typically occur with single-view systems in realistic environments.

The proposed study has three main aims. First, it seeks to present and technically describe an updated version of SANE that integrates dual-depth sensing, higher-frame-rate real-time processing, and an expanded set of clinically relevant spatiotemporal and angular gait parameters, building on earlier work in which SANE was validated against marker-based gold-standard systems. Second, it aims to evaluate the robustness of this dual-depth, real-time marker-less pipeline under realistic operating conditions, by analysing four sessions in a single, clinically examined healthy adult (80 walks that includes full system shutdowns and a 1-week interval between selected sessions. Third, it quantifies robustness via session means, within-session standard deviations, and Relative Error Measurement (REM%) between sessions, and interprets these metrics in relation to published normative ranges for healthy adult gait.

Multi-subject and multi-rater experiments against marker-based systems have already been stablished in our previous work (Chaparro-Rico and Cafolla, 2020; Sipari et al., 2022).

In conclusion, while traditional marker-based gait analysis systems have long been considered the gold standard for detailed biomechanical assessment, their high cost, complex setup, and reliance on post hoc analysis limit widespread and routine use. By contrast, emerging AI-driven, marker-less systems offer substantial advantages in automation, affordability, and accessibility, provided that they can demonstrate accuracy and reliability comparable to established laboratory solutions. The SANE (eaSy gAit aNalysis systEm) platform was originally developed to address exactly this gap by providing an automated, marker-less alternative to labour-intensive marker-based workflows, using depth cameras and AI-based pose estimation to extract clinically relevant gait parameters in a more pragmatic way.

Previous work has already established the clinical validity of SANE. Chaparro-Rico and Cafolla (2020) reported test–retest, inter-rater, and intra-rater reliability for spatiotemporal parameters obtained with SANE in multi-subject protocols, while Sipari et al. (2022) demonstrated strong agreement between SANE and a commercial marker-based system in both laboratory and clinical settings. Together, these studies show that SANE can reproduce standard gait metrics within accepted ranges for clinical and research applications and support its use as a trustworthy measurement tool relative to gold-standard systems.

The present paper builds directly on this validated foundation and has a different but complementary focus. Rather than re-running a full multi-participant clinical validation, we conduct a single-participant, multi-session robustness and system-characterisation study of the latest dual-depth, real-time SANE implementation. Specifically, we investigate how stable SANE’s outputs remain over repeated sessions separated by complete system shutdown and restart, and by a 1-week interval, under realistic operating conditions that reflect typical clinical or laboratory use.

This study has three main aims. First, to present and technically describe the updated SANE system, which integrates dual-depth sensing, higher-frame-rate real-time processing, and an expanded set of spatiotemporal and angular gait parameters, building on earlier work in which SANE was validated against marker-based gold-standard systems. Second, to evaluate the session-to-session robustness of this dual-depth, real-time marker-less pipeline under realistic operating conditions by analysing four sessions (80 walks) in a single, clinically examined healthy adult, each separated by full system shutdown and restart, including a 1-week test–retest interval. Third, to quantify measurement stability using session means, within-session standard deviations, and Relative Error Measurement (REM%) between sessions, and to interpret these indices in relation to published normative ranges for healthy adults.

In this paper, we express reliability and robustness through REM% between session means and within-session standard deviations, which provide direct and interpretable measures of session-to-session percentage differences and dispersion that are well aligned with the technical question addressed here. Multi-subject and multi-rater validation of SANE against marker-based systems has already been reported in our previous work and is therefore not repeated in this study.

## 2 Materials and methods

The SANE (eaSy gAit aNalysis system) platform provides a marker-less, AI-powered approach to human gait analysis, and it is distinguished by its rigorous validation against gold-standard biomechanical methods. In previous clinical studies, SANE’s outputs were compared directly to a reference marker-based vision system (BTS), long considered the gold standard in laboratory gait analysis. This side-by-side evaluation established SANE’s ability to accurately estimate spatiotemporal gait parameters, such as step length, cadence, and gait speed, within clinically acceptable margins of error, establishing the system’s value as a trustworthy measurement tool for both research and rehabilitation.

However, while this initial validation focused on a core set of gait metrics and required post hoc data processing, the latest version of SANE, presented in this paper, radically extends both the breadth and immediacy of its analytic capabilities. Now, the system features true *real-time analysis*, enabling clinicians and researchers to observe gait parameters, visual markers, and 3D reconstructions dynamically, as the person walks. Alongside this leap in temporal performance, reflected in the increase from *15 FPS* in legacy versions to as much as *80 FPS* in the improved system, the software also supports a much broader spectrum of clinical variables.

With its enhanced algorithms, SANE can now quantify not only basic spatiotemporal metrics but also *step and stride length, stride and step time, cadence, step width, individual left/right foot angles, double support time, and distinct swing and stance phases* of the gait cycle. Each metric is computed mathematically from the system’s synchronized dual-camera depth + skeletal streams, using robust geometric and kinematic principals. Finally, the system is able to merge the depth cloud of the cameras to show a 3D merged mesh.

Below, each metric is described with its governing mathematical equation done by each camera, showing the analytic transition from marker-based to modern marker-less, depth camera-driven automatic gait analysis.

### 2.1 Step length $\left(L_{\text{step}}\right)$

Step length quantifies the distance between the point of initial contact of one foot and that of the opposite foot. For consecutive heel-strike events involving the left $p_{L}$ and right $p_{R}$ ankles, this is given by:

$$\left.L_{step}=\left|p_{ankle,right}^{n}-p_{ankle,left}^{n-1}\right.\right|$$

where $p=\left(x,y,z\right)$ is the 3D position at the relevant event frame.

### 2.2 Stride length $\left(L_{stride}\right)$

Stride length is the distance covered between two successive contacts of the same foot:

$$\left.L_{stride}=\left|p_{ankle,right}^{n+1}-p_{ankle,right}^{n}\right.\right|$$

where n indexes distinct strides.

### 2.3 Step width $\left(W_{step}\right)$

The step width represents the mediolateral separation between the feet at initial contact. If the laboratory’s mediolateral direction is the x -axis, then step width at time t is:

$$W_{step}=\left|x_{ankle,right}^{t}-x_{ankle,left}^{t}\right|$$

An average can be computed over all frames in double-support for each stride cycle.

### 2.4 Stride time $\left(T_{stride}\right)$ and step time $\left(T_{step}\right)$

Stride time is the elapsed time between two consecutive heel strikes of the same foot:

$$T_{stride}=t_{HS,right}^{n+1}-t_{HS,right}^{n}$$

Step time refers to the interval between alternating heel strikes:

$$T_{step}=t_{HS,right}^{n}-t_{HS,left}^{n}$$

where $t_{HS}$ signifies the timestamp in seconds at detected heel strike.

### 2.5 Cadence $\left(\;C\;\right)$

Cadence is computed as the number of steps per minute, derived from $T_{step}$ :

$$C=\frac{60}{\bar{T_{step}}}$$

with $\bar{T_{step}}$ the mean step time over all cycles.

### 2.6 Gait speed $\left(\;v\;\right)$

Gait speed, namely, the average walking speed, is found by dividing stride length by stride time:

$$v=\frac{\bar{L_{stride}}}{\bar{T_{stride}}}$$

### 2.7 Left and right foot angle $\left(\theta_{foot}\right)$

Foot angle assesses the orientation of the foot relative to the direction of movement, often orthogonal to gait trajectory. For a foot with heel h and toe t landmarks, and average walking direction d , the angle is:

$$\theta_{foot}=\arccos\left(\frac{\left(t-h\right)\cdot d}{\left|t-h\right.\left|\cdot \left|d\right.\right|}\right)$$

Reported separately for left and right foot at each foot-flat event.

### 2.8 Double support time $\left(T_{DS}\right)$

Double support is defined as the period within a gait cycle when both feet are in contact with the ground. Detected by examining the vertical depth position of each foot, it is given by:

$$T_{DS}=t_{FO,left}^{n}-t_{FS,right}^{n}$$

where $t_{FS}$ is foot-strike and $t_{FO}$ is foot-off for successive steps.

### 2.9 Swing and stance phases $\left(T_{swing},T_{stance}\right)$

Swing phase duration is the time from toe-off to the subsequent heel-strike for a given limb:

$$T_{swing}=t_{HS,right}^{n+1}-t_{TO,right}^{n}$$

Stance phase covers the period from heel-strike to toe-off:

$$T_{stance}=t_{TO,right}^{n}-t_{HS,right}^{n}$$

Relative durations as a percentage of the gait cycle:

$$\%T_{swing}=\frac{T_{swing}}{T_{stride}}\times 100$$

$$\%T_{stance}=\frac{T_{stance}}{T_{stride}}\times 100$$

### 2.10 Automatic gait analysis procedure

In SANE, all temporal metrics depend on precise and automatic event detection*, the automatic step detection*.

After deriving a relevant signal (such as ankle-to-ankle 3D distance) across frames, SANE applies a Gaussian smoothing filter, which attenuates high-frequency noise. The smoothed signal $y_{smooth}\left[n\right]$ at sample n is:

$$y_{smooth}\left[n\right]=\sum_{k=-K}^{K}y\left[n+k\right]\cdot \frac{1}{\sqrt{2\pi }\sigma }\exp\left(-\frac{k^{2}}{2\sigma^{2}}\right)$$

where σ determines the width of the smoothing kernel.

The algorithm to find peaks is then employed to identify local maxima corresponding to biomechanical events such as heel strikes. Mathematically, a peak at $n_{0\;}$ satisfies:

$$y_{smooth}\left[n_{0}\right]>y_{smooth}\left[n_{0}-w\right]\text{ and }y_{smooth}\left[n_{0}\right]>y_{smooth}\left[n_{0}+w\right]$$

for all $\left(\;w=1,\ldots ,W\;\right),\text{with }a\text{ window }W$ tailored to physiological minimum step times related to cadence, in the experimental campaigned presented, in this paper guided by a *70-bpm* metronome.

Each event’s timestamp is then used in all parameter formulas above, creating a seamless pipeline from marker-less observation. These peaks correspond to key gait events, such as heel strikes, occurring at the maximum stride separation, and their detection is the backbone of accurate gait parameters calculation.

At each instant, as the participant advances along the prescribed walkway, dual depth cameras collect RGB and depth data streams. This raw data is processed frame-by-frame to generate smoothed time series for determining the needed features. In addition, cameras pipelines are managed in parallel to threading in individual ports and received in real-time at the same time and then matched. Furthermore, if no data is received from both camera at the same time no computation is performed.

A core advance of the SANE system is its *dual-depth camera architecture*. Where traditional approaches may suffer from occlusion or reduced spatial accuracy due to a single point of view, SANE joins two spatially-calibrated Intel RealSense cameras, each producing an independent 3D measurement of key anatomical landmarks per frame.

In the SANE system, real-world gait parameters and 3D visualizations are produced by accurately mapping 2D landmark detections from synchronized dual depth cameras into a unified, metric laboratory reference frame. Each camera first transforms detected 2D pixel coordinates $\left(x,y\right)$ and their associated depth d into local 3D coordinates by:

$$X=\left(x-c_{x}\right)\frac{d}{f_{x}}$$

$$Y=\left(y-c_{y}\right)\frac{d}{f_{y}}$$

Z=d

where fx,fy are the camera’s focal lengths and cx,cy are its optical centre, as directly obtained from hardware calibration at session start.

**FIGURE 1:**
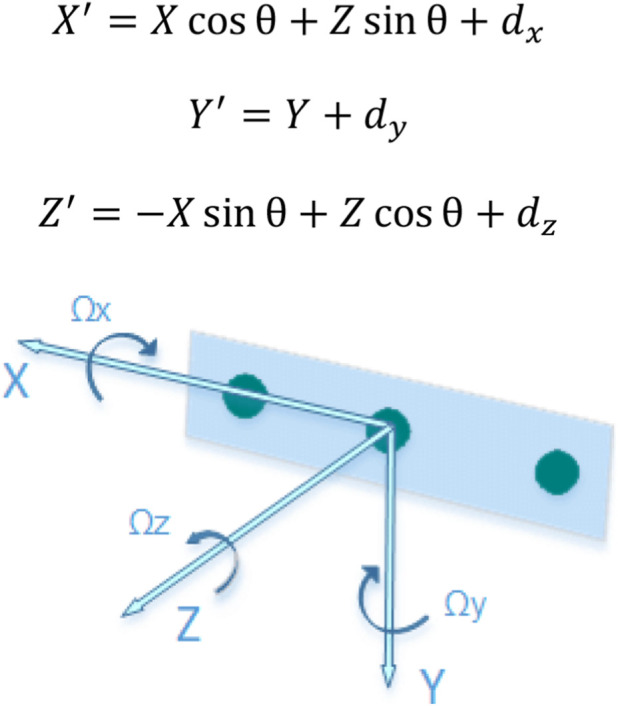
Intel RealSense D435i reference axes (New Technologies, 2019). © Copyright The <librealsense2> Contributors. License: Apache License, Version 2.0.

To reconcile the data from both cameras, which are physically offset and angled relative to each other, SANE applies a rotation of angle θ about the vertical Y−axis (Referred to Reference axes in Figure 1) and a translation defined by the measured displacement dx,dy,dz between cameras. In code and algebraically, for every 3D point from Camera 2, the following transformation is applied:

This step faithfully reconstructs every anatomical landmark from Camera two into the coordinate frame of Camera 1. After transformation, SANE performs landmark fusion: for each joint at each frame, the software selects measurements from both cameras and either averages them or takes the most confident (least occluded) value, yielding a single robust 3D position per landmark.

With all joint coordinates now unified and expressed in meters, SANE calculates gait metrics using the classic Euclidean distance:

$$d=\sqrt{{\left(X_{2}^{*}-X_{1}^{*}\right)}^{2}+{\left(Y_{2}^{*}-Y_{1}^{*}\right)}^{2}+{\left(Z_{2}^{*}-Z_{1}^{*}\right)}^{2}}$$

Here, $\left(X_{1}^{*},Y_{1}^{*},Z_{1}^{*}\right)$ and $\left(X_{2}^{*},Y_{2}^{*},Z_{2}^{*}\right)$ are the fused, globally-aligned 3D coordinates at two gait events, left and right heel strike, or successive steps of the same foot).

**FIGURE 2:**
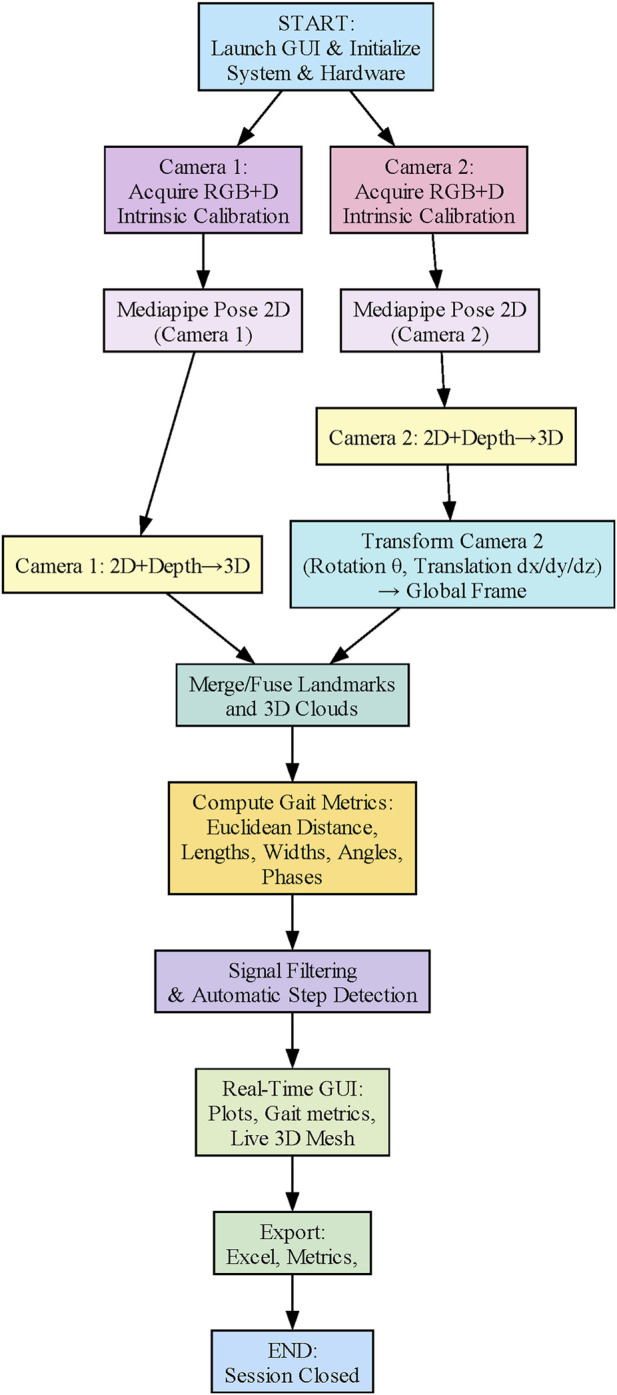
Workflow of the SANE gait analysis system.

The complete workflow of the SANE gait analysis system is summarized in Figure 2, illustrating the dual-camera acquisition branches, point fusion, metric computation, and real-time visualization and export.

In addition to scalar gait parameters, the software uses this same transformation and merging schema to create a *composite 3D point cloud map* by aligning the raw depth images from both cameras, displaying a merged mesh of the person walking in real space in the GUI. This interface not only may support transparent quality control and anatomical assessment by clinicians and researchers, but may also enable frame-by-frame playback and exploration of complex movement patterns.

The real-time alignment and merging of dual depth streams allow SANE to overcome line-of-sight occlusion, enhances confidence in anatomical measurement, and enables the use of 3D mesh data for advanced research such as future studies and implementation on step path profiling, symmetry analysis, or automated detection of abnormal gait. Additionally, it may further open numerous possibilities in, rehabilitation robotics, and clinical follow-up, as all output metrics and meshes are referenced to the true real-world environment and can be compared longitudinally or across populations.

**FIGURE 3:**
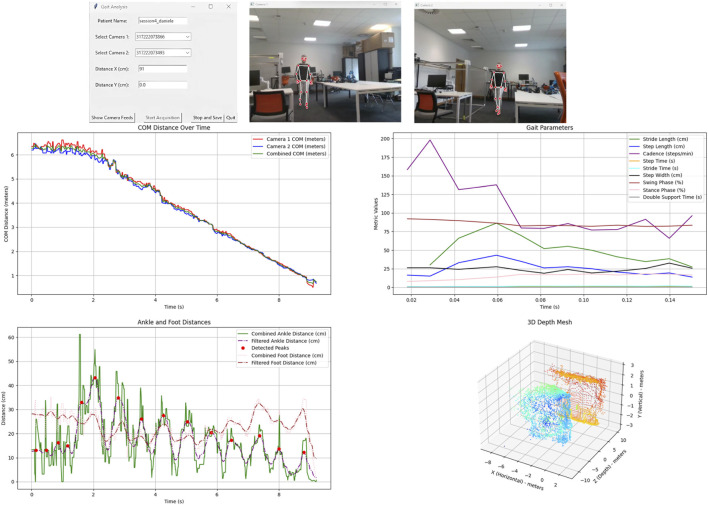
SANE GUI.

Figure 3 showcases the SANE gait analysis system’s graphical user interface (GUI) and its real-time analytical capabilities, giving readers an integrated view of both data acquisition and live biomechanical computation. Below a detailed description of the GUI.

#### 2.10.1 Top row

- On the top left, the GUI control panel allows the user to input the patient’s name and select which RealSense cameras to use for the session. The user can also enter the spatial distances between cameras (needed for accurate 3D fusion), and use buttons to show live camera feeds, start/stop recording, save the session, or exit the application.

The top centre and top right panels present the real-time camera feeds, each with an overlay of Google Mediapipe’s pose estimation skeleton on the subject. Mediapipe BlazePose GHUM Lite has been used to allow real-time pose estimation, threshold are set automatically depending on gait pace.

- This visual feedback ensures that the subject is correctly tracked by both cameras throughout the session, and that the system is receiving quality 2D and 3D joint data for downstream calculations.

#### 2.10.2 Middle row

- The *COM Distance Over Time* plot (middle left) tracks the movement of the subject’s centre of mass (COM) as computed from each camera and from the fused 3D data, allowing users (clinicians or researchers) to monitor the subject’s approach toward the cameras in real-time. Close agreement across traces confirms correct calibration and fusion.
- The *Gait Parameters* plot (middle right) charts numerous walking metrics (stride length, step length, cadence, times, widths, swing/stance/double support phases) over time, providing immediate feedback on gait quality. These metrics are computed live from the dual-camera marker-less measurements using the algorithms described.

#### 2.10.3 Bottom row

- The *Ankle and Foot Distances* graph (bottom left) shows the progression of joint separations with both raw (green) and filtered (purple, pink) curves, alongside red markers at detected peaks, which indicate step events. This step/peak detection is done in real-time and allows for the calculation of spatiotemporal gait metrics.
- The *3D Depth Mesh* plot (bottom right) is a point cloud visualization generated by merging depth data from both cameras, spatially aligned and color-coded. It gives a 3D representation of the subject and allows clinicians to visually validate correct body tracking and fusion in real space.

Users may use the GUI to set up a new session, select hardware, launch live camera views, and begin acquisition with one click, the system has a delay to allow the user if alone to go for performing the walk and start when the full figure is visible only. The GUI provides instant visual assurance that the person is being tracked accurately and that cameras are functioning. When the session is ended, all data and plots can be exported for further analysis or record-keeping.

Users may directly view gait asymmetries, check for missed steps or posture irregularities, and validate the depth/3D mapping of the subject as acquired. Real-time feedback means any protocol issues (e.g., tracking loss, poor calibration) are visible immediately.

In addition, users may use these displays for subject screening, and robust data collection, while the same metrics and visuals facilitate direct comparison with traditional marker-based systems.

### 2.11 Inclusion criteria

The participant was required to be an adult with a healthy, unimpaired gait, free from known neurological, musculoskeletal, or other conditions that could affect walking, and able to ambulate independently without assistive devices. The individual also needed to be capable of understanding and following the metronome-paced walking instructions and of completing repeated trials along the measurement walkway. Participation took place under Swansea University ethical approval number: 1 2025 11492 11921.

### 2.12 Experimental campaign

**FIGURE 4:**
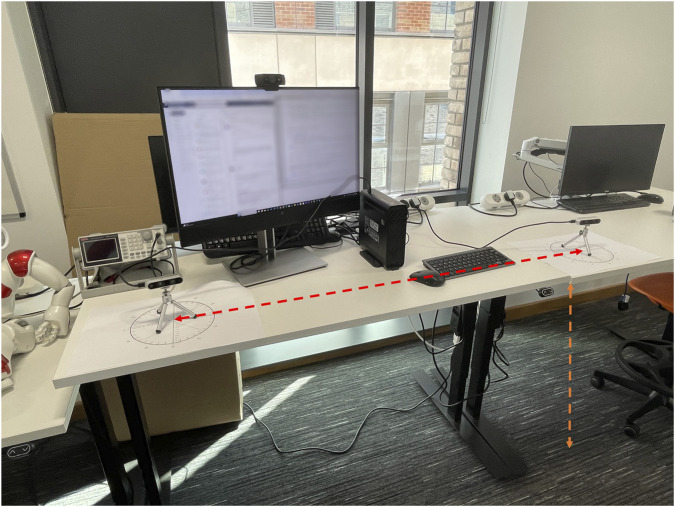
Laboratory camera setup for SANE gait analysis.

**FIGURE 5:**
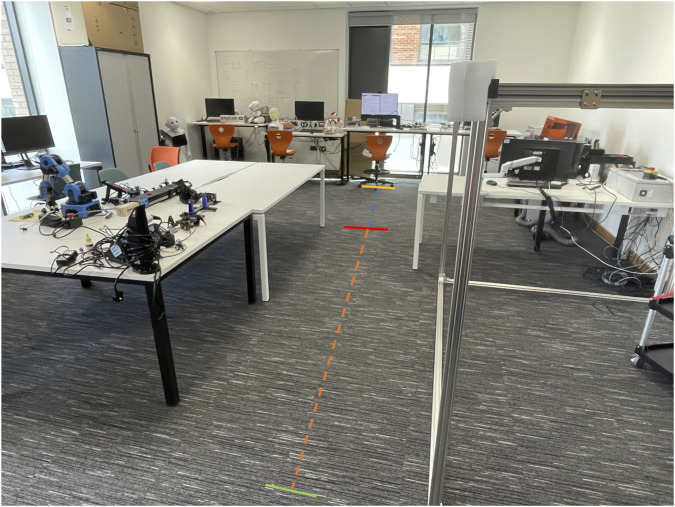
Walking path setup for SANE gait analysis.

The experimental protocol consisted of four sessions; each conducted with the SANE system configured as described above. Figures 4, 5 illustrate the complete laboratory setup and walking protocol used with the SANE dual-depth camera system. As depicted in Figure 4, two Intel RealSense cameras were positioned while Figure 5 the walking path details.

For each session, 20 walking performed by the first author, aged 39, 1.74 m height 73.00 kg weight in healthy condition trials were performed (80 in total), with each walk following a standardized protocol: the participant began at the starting line (Figure 5, green), walked a measured distance of 4.035 m along the marked path (Figure 5, orange line) toward the stop line (Figure 5, red line). The blue line marks an additional 3.741 m from the camera plane to the end of the walking corridor, used for post-walk tracking and safety. During each trial, the participant was explicitly instructed to walk in step with an external electronic metronome set at 70 beats per minute, rather than at their freely chosen or preferred pace. This ensured a standardized walking cadence for all experimental sessions.

As shown in Figure 4, the two depth cameras were spaced 0.910 m apart (Figure 4, red line), fixed at a height of 0.35 m above the floor (Figure 4, orange line), and oriented 20° inward to optimize coverage and minimize occlusion of lower-limb markers. The cameras were installed at a height of 0.35 m to optimize the vertical angle for lower-limb and foot trajectory capture, while the 0.910-m camera spacing was chosen after calibration and coverage testing to provide an ideal baseline for depth fusion and minimize tracking occlusion in our laboratory context. Although these values offered optimal visual coverage for the current experimental environment, the SANE software allows users to set custom camera distances in the interface, supporting flexibility and reproducibility across different scenarios.

Following each session, session duration and timing were logged.

- Session 1: 39 min, 25 s
- Session 2: 27 min, 23 s
- Session 3: 29 min, 35 s
- Session 4: 28 min, 48 s

The design of the experimental campaign specifically addresses two types of reliability commonly assessed in biomechanical validation studies:

Sessions one and two were performed consecutively on the same day, by the same operator using the identical hardware configuration, with a complete system shutdown and re-initialization between sessions. This allows the assessment of the system’s reliability under identical conditions, capturing any effects of system restart or calibration on repeated measurements of gait parameters.

Sessions three and four were conducted *1 week after* the initial two sessions, again each separated by a complete system shutdown and restart. By replicating the full experimental procedure after a temporal delay, this allows evaluation of the SANE system’s stability and measurement reproducibility across different days, capturing variability due to long-term configuration drift, environmental changes, or subtle differences in participant condition.

The background environment, as shown in the experimental setup figures, was intentionally not strictly controlled in order to reflect real-life clinical scenarios. Throughout all testing, no adverse effects on parameter accuracy or reliability were observed due to background objects, confirming the robustness of the dual-depth SANE workflow to environmental variability.

This experimental approach ensures rigorous validation of the SANE system both within the same testing day (intra-rater), and across a clinically typical time window (test–retest), in line with best practices in gait analysis research.

### 2.13 Variation between sections

To quantitatively assess the variation between sessions, the *Relative Error Measurement (REM%)* have been computed (reported in Table 1) for both intra-rater and test–retest experiments. REM% is defined as:

$${\text{REM}\%}_{A,B}=\frac{\left|{\bar{X}}_{A}-{\bar{X}}_{B}\right|}{\left|{\bar{X}}_{A}\right|}\times 100$$

where ${\bar{X}}_{A}$ and ${\bar{X}}_{B}$ are the mean values of a given gait parameter in sessions A and B, respectively. For the intra-rater experiment, REM% was calculated for session 1 vs. 2 (same day and consecutively) and session 3 vs. four (1 week later, but same protocol/operator). For the test–retest experiment, REM% was computed for session 2 vs. three (1-week interval between sessions).

**TABLE 1:** Relative Error Measurement (REM%) for the intra-rater and test–retest experiments.

Metric	REM% session 1 Vs. session 2 (intra-rater)	REM% session 2 Vs. 3 session (test–retest)	REM% session 3 Vs. session 4 (intra-rater)
Gait speed	3.11	4.34	2.30
Step length	2.40	4.13	3.6
Stride length	4.85	4.13	2.01
Cadence	4.67	4.98	3.94
Step time	0.71	2.26	2.25
Stride time	1.89	1.08	2.25
Step width	3.53	5.29	3.04
Left foot angle	3.09	1.12	4.88
Right foot angle	4.94	0.29	1.19
Double support time	3.77	6.91	3.63
Swing phase	0.30	3.61	0.58
Stance phase	1.32	5.03	6.30

## 3 Results

To assess reliability of the SANE marker-less gait analysis system, four experimental sessions were performed, each consisting of 20 standardized walking trials (see Methods). Sessions one and two were conducted consecutively on the same day *(intra-rater test)*, with a full system shutdown and re-initialization between runs. Sessions three and four were performed *1 week later* under identical conditions, again with a system restart between them (*test–retest)*.

Table 2 presents, for each session, the mean ± standard deviation (SD) of the computed gait parameters: gait speed, step length, stride length, cadence, step time, stride time, step width, left and right foot angles, double support time, swing phase, and stance phase.

**TABLE 2:** Mean ± standard deviation of measured gait parameters for eachsession.

	Gait speed (cm/s)	Step length (cm)	Stride length (cm)	Cadence (steps/min)	Step time (s)	Stride time (s)	Step width (cm)	Left foot angle (°)	Right foot angle (°)	Double support time (s)	Swing phase (%)	Stance phase (%)
Session 1	139.11 ± 28.52	29.38 ± 1.88	57.40 ± 3.76	107.20 ± 13.28	0.64 ± 0.06	1.28 ± 0.12	23.88 ± 1.09	1.41 ± 6.75	1.71 ± 6.23	0.15 ± 0.04	81.63 ± 1.90	18.37 ± 1.90
Session 2	143.43 ± 37.64	30.09 ± 1.54	60.18 ± 3.08	102.20 ± 16.34	0.64 ± 0.05	1.30 ± 0.09	23.04 ± 0.94	1.45 ± 6.82	1.63 ± 8.45	0.16 ± 0.02	81.39 ± 1.30	18.61 ± 1.30
Session 3	149.66 ± 36.62	31.33 ± 3.67	62.67 ± 7.34	107.28 ± 12.36	0.66 ± 0.05	1.31 ± 0.11	21.82 ± 1.43	1.43 ± 6.27	1.62 ± 5.57	0.15 ± 0.03	84.33 ± 1.36	17.67 ± 1.36
Session 4	153.10 ± 39.34	32.46 ± 4.82	63.93 ± 9.64	103.06 ± 13.98	0.67 ± 0.08	1.34 ± 0.17	22.48 ± 1.71	1.50 ± 6.33	1.64 ± 7.85	0.14 ± 0.02	83.84 ± 2.06	16.56 ± 2.06

Overall, these REM% results demonstrate low session-to-session variability across all primary gait metrics, with the vast majority of REM% values below 5%. The SDs were low and consistent with published norms. REM% and SDs support a high level of measurement reliability and repeatability when using the SANE system. Comprehensive interpretation and comparison to clinical reference standards are provided in the Discussion section.

In Table 1, only a small subset of phase-related parameters show REM% values above 5%: double support time reaches 6.91% in the week-apart comparison, and stance phase reaches 5.03% and 6.30% in two inter-session comparisons, while all other parameters (gait speed, step/stride length, cadence, step/stride time, step width, foot angles, swing phase) remain below or around 5%. We interpret REM% values up to approximately 6%–7% for stance phase and double support as reflecting normal physiological variability. Changes in gait kinematics of greater than approximately 5° in young healthy adults were also identified when comparing between sessions using other systems in related works (Wilken, 2012).

**FIGURE 6:**
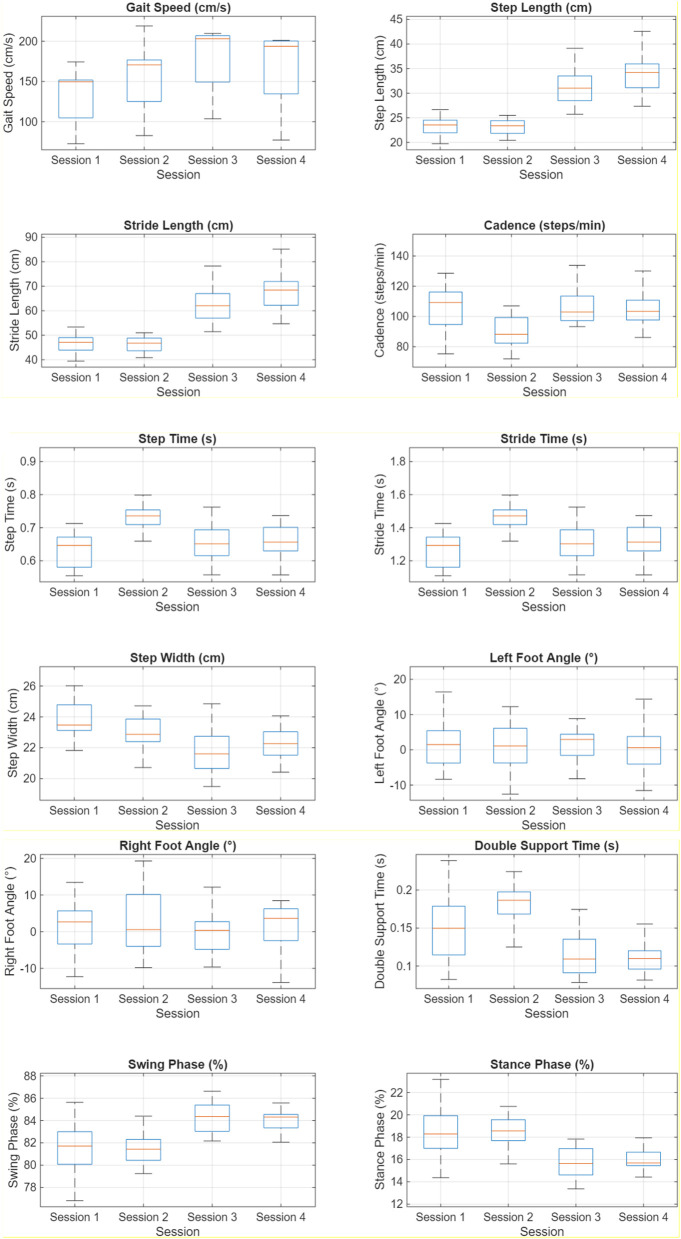
Boxplots of representative gait parameters across the four acquisition sessions. Boxplots of gait parameters across the four sessions (20 walks per session, 80 walks total).

In addition to the numerical indices in Tables 1, 2 and Figure 6 provides a distribution-level view of the gait parameters across the four acquisition sessions. For the primary spatiotemporal metrics (Figure 6A), median gait speed increases modestly from around 1.5 m/s in Session one to just under 2.0 m/s in Sessions three to four, with interquartile ranges covering roughly 1.2–1.6 m/s in the first session and 1.7–2.1 m/s in the later sessions; all values remain within a clinically typical range of approximately 0.8–2.2 m/s. Step and stride length medians follow a similar pattern, rising from about 22 to 24 cm and 45–50 cm to around 33–35 cm and 65–75 cm respectively, while the spreads of the distributions remain comparable between sessions. Cadence medians stay within a relatively narrow band of about 95–110 steps/min, with interquartile ranges typically between ∼85 and 120 steps/min and no session exhibiting markedly larger variability. These overlapping interquartile ranges and limited median shifts are consistent with the low REM% values for the primary spatiotemporal parameters and indicate that, despite small day-to-day differences in walking pattern, the dual-depth SANE configuration yields stable spatiotemporal estimates across sessions.

The temporal and support parameters in Figure 6B (step time, stride time, step width) exhibit even tighter distributions. Step and stride time medians lie between approximately 0.60–0.70 s and 1.25–1.45 s, respectively, with narrow boxes and short whiskers reflecting small within-session dispersion. Step width medians are centred near 22–24 cm in all sessions, with most values falling in the 20–26 cm range. This behaviour matches the very low SDs and REM% reported numerically and supports the conclusion that SANE’s event timing and lateral support estimates are highly consistent over repeated shutdown and restart cycles. Angular and phase-related parameters in Figures 6B,C (left and right foot angles, double support time, swing and stance phases) naturally display greater spread: foot angles are centred near neutral (medians around 0°–3°) but span approximately −10° to +15°, double support time medians range from about 0.11 to 0.20 s, and swing and stance phase medians vary within a few percentage points around ∼81–86% and 14%–19%, respectively. These broader boxes and slightly larger median differences between sessions are in line with the higher SDs and REM% (up to about 6%–7%) obtained for these variables; however, their distributions remain within expected healthy bounds and show no systematic drift from early to late sessions. Overall, the ranges observed in Figure 6 corroborate the REM% and SD analyses and indicate robust session-to-session behaviour of the SANE system for all reported gait parameters in this single-participant study.

## 4 Discussion

This study provides an in-depth evaluation of the marker-less SANE gait analysis system, which was initially launched in 2020 and initially tested in laboratory environments with healthy subjects; it was subsequently validated in clinical settings against gold-standard marker-based motion capture and expanded in 2022 with enhanced capabilities.

SANE was previously validated in direct comparison with marker-based gold-standard systems across core gait parameters, see (Chaparro-Rico and Cafolla, 2020; Sipari et al., 2022). In the present study, we extend this foundation by stress-testing SANE’s robustness, session-to-session repeatability, expanded metric suite, and real-time workflow in realistic clinical conditions and over multiple repeated sessions. Here we focus on robustness across four sessions including shutdown/restart, using REM% and within-session SD, our results show that SANE is operationally resilient and stable across sessions, supporting its use in challenging clinical environments.

The latest version of SANE, as demonstrated here, implements and systematically tests a set of expanded, clinically relevant gait parameters, all enabled by dual-depth, real-time processing architecture and a robust, reproducible experimental campaign.

The advantage of SANE’s dual-camera approach, as compared with most single-camera systems, arises from its continuous capture of all anatomical landmarks—even in realistic, cluttered, or occlusion-prone environments. Unlike other marker-less or depth-based systems reported in recent literature, which can suffer from frequent tracking loss or parameter dropouts when a limb becomes hidden, SANE’s dual-field fusion sustains accurate 3D localization throughout the entire gait cycle, ensuring reliable collection of all spatiotemporal and angular metrics relevant for robust clinical decision-making. This operational advantage was experimentally confirmed in our multi-session, multi-environment protocol as demonstrated by the absence of tracking failures across 80 gait trials.

The SANE system autonomously quantifies a comprehensive suite of spatiotemporal and kinematic parameters, computed from synchronized 3D camera measurements and AI-driven body landmark detection. Throughout the four-session campaign, these outputs included *gait speed*, *step length*, *stride length*, and *cadence*, but also *step time*, *stride time*, *step width*, *left and right foot angles*, *double support time*, *swing phase (%)*, and *stance phase (%)*; each mapped frame-by-frame to provide a live, multidimensional portrait of gait dynamics.

Examining the sessions, SANE outputs align well with established normative values in the literature (Bohannon, 1997; Whittle, 2014; Burnfield, 2010; Koo and Li, 2016). For this healthy *39-year-old adult* (first author of the paper), the measured *gait speed* ranged from *139.11 to 153.10 cm/s*, which sits squarely within published reference intervals for adults in their fourth decade, typically cited as *125–150 cm/s* for preferred or fast walking. The *step length* spanned *29.38–32.46 cm*, and *stride length* ranged *57.40–63.93 cm*, which are remarkably close to values established for healthy adult. *Cadence* (mean: *102.20–107.28 steps/min*), *step time* (*0.64–*0.67 s), and *stride time* (*1.28–*1.34 s) all fall within predicted normative ranges for this cohort. The *step width* (*21.82–23.88 cm*) is indicative of a stable gait base, while the *left and right foot angles* (approximately *1.4–1.7 degrees* each) document the absence of asymmetry or notable toeing abnormalities, further confirming technical and anatomical validity.

Temporal and phase-related metrics also reflected characteristic distributions for a healthy subject walking at a brisk pace, with *swing phases* of *81%–84%*, *stance phases* of *16%–19%*, and *double support times* between *0.14–*0.16 s. Each of these parameters was computed automatically and in real-time. All of this shows that the SANE system may provide clinicians and researchers immediate, detailed insight into subject performance as acquisition unfolded.

A central strength demonstrated by this work is the strong *reliability* of the SANE platform, as quantified by the *Relative Error Measurement (REM%)* across all parameters for intra-rater and test-retest experiments. The REM% for *gait speed ranged from 2.30% to 4.34%*, while analogous values for *step length* (*2.40%–4.13%*), *stride length* (*4.85%–4.13%*), and other parameters likewise remained well below or around *5%*, even across sessions separated by a full system reboot or a *1-week* temporal gap. The mentioned reliability results fall within the ranges typically reported for marker-based commercial systems and align closely with the best-practice reliability results found in the literature. While minor increases in REM% for phase metrics (such as *stance phase*, up to *6.90%* after a week) were observed, these deviations remain well within clinically accepted tolerances and may reflect expected day-to-day physiological variation in real patients rather than measurement drift. Notably, the comprehensive SANE system design; integrating dual-camera 3D fusion, explicit rotation/translation calibration, and automatic event detection, proved resilient to both operator manipulation and technical restarts.

Beyond session means and REM%, the standard deviations (SDs) observed for all gait parameters are critical indicators of high repeatability and measurement stability of the SANE system. For most parameters, within-session SDs were low and consistent with published norms in the mentioned literature (Bohannon, 1997; Whittle, 2014; Burnfield, 2010; Koo and Li, 2016) for healthy adult gait, reinforcing both the fidelity of real-time AI-driven joint estimation and the stability of the dual-depth platform.

For example, *gait speed* SDs ranged from *28.52 cm/s* (Session 1) to *39.34 cm/s* (Session 4), all falling within expected ranges for a single healthy subject performing repeated walks, mentioned literature reports SDs between *25 and 35 cm/s* for similar age groups in laboratory conditions, with minor deviations often attributed to slight pace adjustments or walking dynamics across trials. Similarly, *step length* SDs ranged from *1.54 cm* (Session 2) to *4.82 cm* (Session 4), and *stride length* SDs ranged from *3.08 cm* (Session 2) to *9.64 cm* (Session 4). The highest stride length SD in Session 4, at *9.64 cm*, exceeds some laboratory reference norms, typically *5–7 cm*, but remains plausible, particularly when considering potential minor cumulative alignment error, phase-detection variability during long walk series, or natural variation even in healthy individuals as fatigue or attention fluctuates.

Temporal metrics exhibited consistently modest variation: *cadence* SDs ranged from *12.36 steps/*min (Session 3) to *16.34 steps/*min (Session 2), while *step time* and *stride time* SDs were confined to *0.05–*0.08 s and *0.09–*0.17 s, respectively. These values were uniformly comparable to published clinical datasets and to what would be expected for brisk, regular walking in adults.

A few higher SDs emerged in angular parameters, notably *left foot angle* (*6.27–6.82°* in Sessions two and 3) and *right foot angle* (*5.57–8.45* in Sessions two and 4). For instance, the highest value, *8.45°* for right foot angle in Session 2, is above mean healthy-population SDs typically reported as *4–6°*, but within ranges for individual-stride angular variability in real-world walking. Such increased variability may be due to small differences in foot placement with respect to the walkway, slight shifting of attention or balance as sessions progress, or the sensor sensitivity to dynamic off-axis motion, which is a recognized challenge even for marker-based high-speed motion capture.

Composite spatial parameters also showed moderate SD increases in selected sessions; *step width* varied from *0.94 cm* (Session 2) to *1.71 cm* (Session 4)—both lower than the *∼two to three cm* SDs described for healthy adults in repetitive walking, suggesting consistent lateral placement. *Double support time*, *swing phase (%)*, and *stance phase (%)* all remained below or around *2%* SD for all sessions, underscoring highly stable detection of gait cycle events.

The distribution patterns in Figure 6 further substantiate the robustness conclusions derived from the numerical analysis. For gait speed, step and stride length, and cadence, the boxplots demonstrate substantial overlap of interquartile ranges across the four sessions, with medians confined to relatively narrow intervals (approximately *1.5–*2.0 m*/s* for speed, *22–35 cm* for step length, *45–75 cm* for stride length, and *95–110 steps/*min for cadence). These values are well within normative healthy ranges and the modest differences between sessions are consistent with the small REM% values reported for these metrics, suggesting that the observed variations largely reflect normal day-to-day changes in the participant’s walking pattern rather than instability in the measurement system. The very tight boxes for step and stride time and step width, with nearly superposed medians across sessions, mirror the low SDs and REM% for these parameters and indicate that SANE provides highly repeatable estimates of temporal and support characteristics under repeated shutdown and restart conditions. Although angular and phase-related parameters (foot angles, double support, swing and stance phases) exhibit comparatively wider distributions and slightly larger shifts in central tendency—behaviour that is expected given their greater intrinsic variability—their medians remain within expected healthy bounds (foot angles near neutral, swing and stance around *80%–86%* and *14%–19%)* and do not display a monotonic trend over sessions. We therefore interpret the additional spread in these measures primarily as inherent step-to-step and day-to-day variability, rather than as an indication of system drift, and conclude that the dual-depth SANE implementation is operationally robust across the four sessions studied, particularly for the core spatiotemporal and temporal gait parameters.

Presenting and interpreting both lower and higher observed standard deviations demonstrates that SANE accurately reflects genuine physiological variability both within and between walking sessions. The low SDs found for most spatiotemporal parameters indicate repeatable and stable tracking from trial to trial, reinforcing the system’s capability for consistent gait quantification. Where higher SDs were observed were still within plausible physiological limits, likely arising from real step-to-step differences, minor experimental fluctuations, or the challenges of automated angle estimation in natural gait.

REM% for gait speed, step/stride length, cadence, step/stride time, step width, and foot angles was below 5% for all sessions. For stance phase and double-support time, REM% reached 6.3% and 5.2% in some sessions (Table 1). All observed results align with clinical reliability thresholds from (Burnfield, 2010) and marker-based system comparison in (Chaparro-Rico and Cafolla, 2020; Sipari et al., 2022).

Notably, none of the standard deviations reached levels that would obscure clinical interpretation or detract from practical measurement value; session averages and REM% reliability metrics remained robust throughout.

These results highlight a key strength of SANE, which is sensitive enough to quantify detailed, real-world variations in gait, but also fall within ranges typically reported for marker-based and manual reference systems.

While SANE offers clear advantages in automation, marker-less technology, and comprehensive parameterization, its greatest remaining limitation may be the moderate increase in variability for certain angular and composite measures. Nonetheless, these findings support the use of SANE for both research and clinical gait assessment in healthy adults, with caution recommended when interpreting individual stride-based angles in populations with highly atypical gait patterns.

The present work has limitations that will be addressed in future studies, although these do not compromise the validity of the current findings. This study’s single participant, repeated measures design was performed to assess the operational robustness of the SANE system under realistic conditions. Consequently, the findings, while comprehensive for a healthy adult, cannot be directly generalized to broader or pathological populations, to a wider range of walking speeds and tasks, or to different clinical environments. Validation was performed in a single laboratory with a fixed camera arrangement, so the effects of alternative room layouts and lighting conditions remain to be systematically quantified. Future work will therefore extend the evaluation of SANE to larger and more diverse cohorts, including clinical populations with gait impairments and conduct additional concurrent validations against commercial systems. In parallel, planned developments include application within patient groups, expanded home based assessment, integration with wearable or contextual data, and further protocol automation to support single clinician or unsupervised use. Future work will also consider sustainability and deployability aspects (e.g., hardware selection, power consumption, and installation footprint) using structured eco design and innovation frameworks (Lee, 2024).

In clinical practice, SANE’s real-time analytics are designed to complement, not replace, standard post-session analysis reports. The principal clinical utility of real-time processing lies in immediate quality assurance: enabling identification and remediation of session errors during acquisition, reducing preparation and workflow times (see Introduction), and enhancing feedback to both clinicians and patients. This supports more efficient, error-resistant, and engaging clinical workflows—particularly advantageous where traditional marker-based systems are too slow or intensive for routine use.

## 5 Conclusion

The results of this study demonstrate that the latest SANE dual-depth marker-less gait analysis system provides an extended real-time capability with strong session-to-session stability. Though dual-depth cameras, advanced 3D fusion, and AI-driven joint detection, SANE now delivers a comprehensive, real-time suite of spatio-temporal and angular gait parameters, including *gait speed, step and stride length, cadence, step and stride time, step width, individualized left/right foot angles, double support*, and detailed *phase timing*. Notably, all parameters are calculated and visualized live, with an exceptionally high frame rate (FPS), representing a substantial and practical improvement compared to prior SANE versions and most legacy marker-based systems.

The system’s measurement reliability was confirmed through rigorous testing. Across both intra-rater and test–retest protocols, and even after full operational restarts and a week between sessions, relative error measurements (REM%) for all core gait metrics consistently remained below 5%. This confirms that SANE achieves high day-to-day and session-to-session reliability, falls within ranges typically reported traditional laboratory-based solutions, supporting its use for longitudinal monitoring and clinical follow-up. Furthermore, low SDs for most spatiotemporal parameters indicated repeatable and stable tracking from trial to trial, reinforcing the system’s capability for consistent gait quantification.

By enabling live, multidimensional assessment in a practical workflow, without markers, lengthy calibration, or manual post-processing, SANE stands out as an accessible, high-precision tool for modern gait analysis. The system’s innovation in real-time analytics, extended parameterization, high FPS, and demonstrated reliability across all key spatiotemporal and angular metrics position it as an attractive solution for a broad spectrum of clinical, rehabilitation, sports, and research contexts.

Future work will broaden this validation to diverse and pathological populations and continue to evolve SANE’s automatic analytics and usability, but the present findings already support its adoption as a next-generation, reliable, and comprehensive platform for marker-less gait assessment.

## Data Availability

The raw data supporting the conclusions of this article will be made available by the authors, without undue reservation.
